# Community-Acquired Acute Kidney Injury in Central India: Etiology, Clinical Profile, and In-Hospital Outcomes

**DOI:** 10.7759/cureus.107425

**Published:** 2026-04-20

**Authors:** Bharatsing D Rathod, Anurag V Ghotkar, Anand Chellappan, Rajashree S Khot, Sunita Kumbhalkar, Prashant P Joshi

**Affiliations:** 1 General Medicine, All India Institute of Medical Sciences Nagpur, Nagpur, IND; 2 Nephrology, All India Institute of Medical Sciences, Bilaspur, IND; 3 Nephrology, All India Institute of Medical Sciences Nagpur, Nagpur, IND

**Keywords:** acute kidney injury, acute respiratory distress syndrome, chronic kidney disease, community-acquired acute kidney injury, glomerular filtration rate, intensive care unit, kidney disease: improving global outcomes, multiple organ dysfunction syndrome, renal replacement therapy

## Abstract

Background

Community-acquired acute kidney injury (CA-AKI) remains a significant public health challenge, characterized by diverse etiologies and variable outcomes. Despite its burden, prospective data from Central India are scarce. This study aimed to systematically delineate the etiological spectrum, clinical characteristics, and in-hospital outcomes of patients with CA-AKI admitted to a tertiary care center in Central India.

Methods

We conducted a prospective observational study of patients presenting with CA-AKI to a tertiary care center in Central India. Eligible participants were identified at admission based on KDIGO (Kidney Disease: Improving Global Outcomes) criteria, and detailed clinical, laboratory, and etiological data were systematically recorded. Patients were stratified by AKI stage and underlying etiology, with outcomes assessed in terms of renal recovery, complications, and mortality.

Results

Among 264 patients, infections emerged as the leading cause of CA-AKI (142, 53.8%), most frequently respiratory (52, 19.7%), urinary tract (34, 12.9%), and gastrointestinal (25, 9.5%). Sepsis (73, 27.7%) and volume depletion (50, 18.9%) were the predominant mechanisms. The cohort was predominantly men (172, 65.2%) with a mean age of 52.2±16.0 years. Pre-renal AKI was most common (197, 74.6%), followed by intrinsic renal (55, 20.9%) and post-renal (12, 4.5%) disease. Hypertension (98, 37.1%), diabetes mellitus (75, 28.4%), and chronic liver disease (40, 15.2%) were frequent comorbidities. At presentation, Stage 1 AKI predominated (157, 59.5%), with Stage 2 (57, 21.6%) and Stage 3 (50, 18.9%) being less common. Renal replacement therapy was required in 20 patients (7.6%). Recovery was highest in pre-renal AKI (125, 78.1%) and Stage 1 AKI (113, 71%). Overall mortality was 32 (12.1%). Mortality was strongly associated with complications, including multi-organ dysfunction syndrome (odds ratio (OR) 4.56; 95% confidence interval (CI), 1.33-15.66; p=0.016), need for mechanical ventilation (OR 17.3; 95% CI, 5.22-57.19; p=0.001), and encephalopathy (OR 6.32; 95% CI, 1.68-23.83; p=0.006).

Conclusions

Infections represent the most prominent etiology of CA-AKI in Central India, with sepsis and volume depletion as the predominant mechanisms. Patients with pre‑renal AKI and those presenting at stage 1 AKI were more often associated with better renal recovery, underscoring the potential reversibility of early disease when promptly recognized, whereas the development of complications, such as multi-organ dysfunction and respiratory failure, was associated with increased mortality. These findings underscore the urgent need for region-specific strategies that strengthen infection control, improve access to early care, and prioritize aggressive management of complications to reduce the burden of CA-AKI in resource-limited settings.

## Introduction

Acute kidney injury (AKI) is a sudden, often reversible decline in renal function that develops over hours to days, typically marked by a rapid rise in serum creatinine and/or reduction in urine output [1]. It is a common clinical syndrome with a broad etiological spectrum, complicating approximately 5% of hospital admissions and up to 30% of intensive care unit admissions [2].

The pathophysiology of AKI is traditionally classified as pre-renal, intrinsic renal, or post-renal, though considerable overlap exists among the mechanisms of injury. Ischemia, hypoxia, and nephrotoxicity are major precipitating factors, each leading to a rapid fall in glomerular filtration rate (GFR). The resulting impairment of filtration drives retention of nitrogenous waste, extracellular fluid accumulation, and disturbances in electrolyte and acid-base homeostasis [3-5].

The burden of AKI is greatest in low- and middle-income countries, where it contributes substantially to short-term morbidity and mortality, prolonged hospitalization, and escalating health-care costs. Long-term sequelae include chronic kidney disease, kidney failure, cardiovascular complications, and excess mortality [6]. AKI present at or within 48 hours of hospital admission is generally attributed to community-based factors and termed community-acquired AKI (CA-AKI) [7]. In contrast, AKI developing after 48 hours of hospitalization is considered hospital-acquired (HA-AKI), often arising from therapeutic interventions or progression of underlying illness. CA-AKI typically occurs in otherwise healthy individuals following acute insults such as dehydration, infection, trauma, and envenomation (including snake-bite), whereas HA-AKI is more frequently linked to sepsis, drug-induced nephrotoxicity, or hospital-related procedures.

CA-AKI is reported most frequently from resource-constrained regions in tropical and subtropical countries. Meta-analyses suggest that HA-AKI is associated with higher rates of oliguria, increased ICU admissions, and longer hospital stays compared with CA-AKI [8]. Regional studies from India highlight striking heterogeneity in AKI prevalence and etiology: snakebite-related AKI is prominent in certain areas, while infections such as pyelonephritis predominate elsewhere [9-11]. Despite this, prospective data on CA-AKI in Central India remain limited.

We therefore undertook a prospective study to define the etiologies, clinical characteristics, and in-hospital outcomes of CA-AKI in patients admitted to a tertiary care hospital in Central India, applying Kidney Disease: Improving Global Outcomes (KDIGO) criteria to ensure standardized assessment. In addition, we sought to identify predictors of in‑hospital mortality among affected patients. In addition, we sought to identify predictors of in‑hospital mortality among affected patients.

## Materials and methods

Study design and setting

We conducted a prospective observational study between October 2022 and April 2024 in the General Medicine Ward, Medical ICU, and Nephrology ward of a tertiary care hospital in Central India. All consecutive patients aged ≥18 years who were diagnosed with CA-AKI and provided informed consent were enrolled. The study protocol was approved by the Institutional Ethics Committee (IEC/Pharmac/2023/552, dated February 11, 2023), and patient confidentiality was maintained. Participants retained the right to withdraw from the study at any time.

Data sources and definitions

Clinical data were obtained from hospital records, including patient history, physical examination, and laboratory investigations.

Definitions of AKI, AKD, CKD, and Renal Recovery Outcomes

AKI is defined as an abrupt decline in kidney function according to the KDIGO 2012 diagnostic criteria [12]. AKI is diagnosed when there is an increase in serum creatinine of at least 0.3 mg/dl within 48 hours, an increase in serum creatinine to at least 1.5 times the baseline value within seven days, or a reduction in urine output to less than 0.5 ml/kg/h for six hours.

Staging of AKI is based on the severity of these abnormalities: Stage 1 is characterized by a serum creatinine level 1.5 to 1.9 times the baseline or an increase of at least 0.3 mg/dl or urine output less than 0.5 ml/kg/h for six to 12 hours; Stage 2 is defined by a serum creatinine level 2.0 to 2.9 times the baseline or urine output less than 0.5 ml/kg/h for 12 hours or longer; and stage 3 is defined by a serum creatinine level at least 3.0 times the baseline or at least 4.0 mg/dl, initiation of renal replacement therapy, an eGFR less than 35 ml/min/1.73 m² body surface area.

Acute kidney disease (AKD) refers to acute or subacute kidney damage or loss of function lasting from seven to 90 days after an episode of AKI [13]. Staging of AKD is based on serum creatinine relative to baseline: stage 0 is defined as a level less than 1.5 times baseline; stage 1 as 1.5 to 1.9 times baseline; stage 2 as 2.0 to 2.9 times baseline; and stage 3 as at least 3.0 times baseline, a level of at least 4.0 mg/dl, or ongoing renal replacement therapy.

Chronic kidney disease (CKD) is defined as the persistence of kidney disease for more than 90 days.

Renal recovery outcomes are defined by resolution of AKI according to KDIGO criteria. Complete recovery denotes full reversal of AKI, which may occur early (within 48 hours) or in a delayed fashion (between 48 hours and seven days).

For the purposes of classification, baseline creatinine is estimated from the mean outpatient values obtained seven to 365 days before hospital admission.

Etiologies were classified according to predefined clinical and laboratory criteria, supplemented by physician judgment, with independent verification by two investigators. Clinical management protocols - including fluid resuscitation, antibiotic therapy, and criteria for renal replacement therapy - were standardized according to institutional guidelines. Missing data were handled by list-wise deletion when <5% and sensitivity analyses were performed.

Laboratory assessments included serum creatinine, electrolytes (sodium, potassium), liver function tests, hemoglobin, and total leukocyte count. Comorbidities such as diabetes mellitus, hypertension, and chronic liver disease were documented.

Study population

Eligible patients were those aged ≥18 years with CA-AKI diagnosed at admission or within 48 hours of hospitalization. Exclusion criteria included chronic kidney disease, hospital-acquired AKI, and hospitalization exceeding 48 hours prior to AKI onset.

A sample size of 264 patients was calculated based on an expected mortality rate of 22%, a 95% confidence level, and a 5% margin of error [10].

Study procedures

Patients were admitted through the emergency department or outpatient clinics to medicine or nephrology wards and the ICU. Detailed clinical histories were obtained, and comorbid conditions were recorded. Laboratory investigations were performed at admission and monitored during hospitalization. Urine output was measured every six hours on day 1 and every 24 hours thereafter. The specific etiology of CA-AKI was noted. When AKI was multifactorial, the single factor judged by the treating physician as the most contributory factor was attributed to AKI. Sepsis was defined according to the Third International Consensus Definition as a documented source of infection with a quick Sequential Organ Failure Assessment (qSOFA) score ≥2 [14].

The management of CA-AKI followed institutional protocols and KDIGO guidelines, including antibiotics, intravenous fluids, vasopressors, blood products, and nutritional support. Dialysis was initiated when indicated for uremic encephalopathy, hyperkalemia, severe acidosis, or fluid overload. The modality (hemodialysis or slow low-efficiency dialysis) and number of sessions were individualized. Patients were followed until discharge or death.

**Figure 1:**
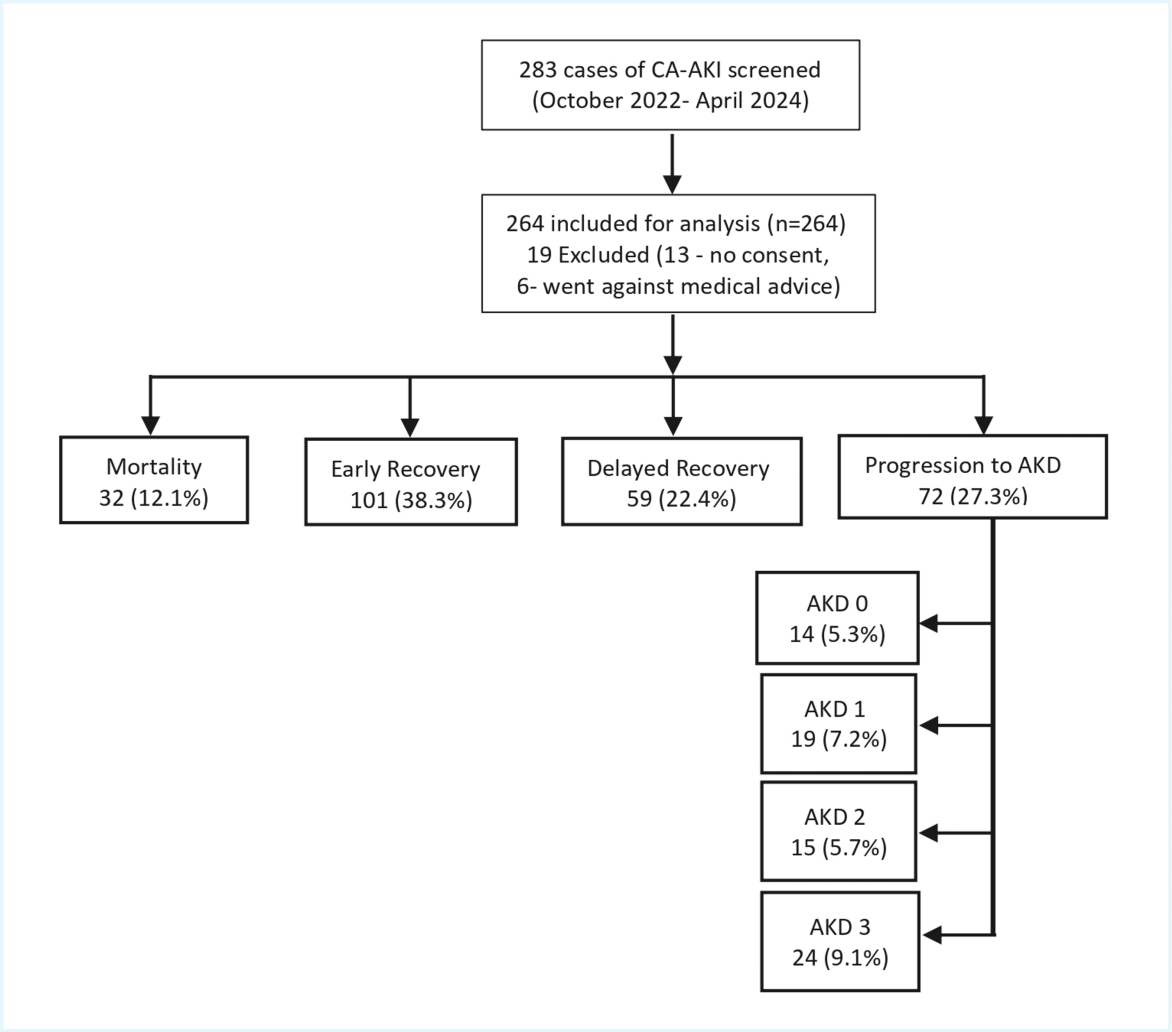
Study flowchart of CA-AKI patients. CA-AKI: Community-acquired acute kidney injury, AKD: acute kidney disease.

Patient outcome(s) were classified as survivor or non-survivor. In survivors, complete recovery (CR) or partial recovery (early or delayed) was noted. A flow diagram illustrating the study process is presented in Figure 1.

Statistical analysis

Data were extracted into a master chart and analysed using descriptive and inferential statistics. Continuous variables (age, duration of illness, hospital stay, ventilator days, biochemical parameters) were expressed as mean±standard deviation (SD) if normally distributed, or as median with interquartile range if skewed. Categorical variables (clinical features, complications, outcomes, dialysis requirement, ventilator support) were presented as frequencies and percentages.

Comparisons between survivors and non-survivors were performed using Student’s t-test for normally distributed continuous variables and the Mann-Whitney U test for non-normally distributed data. Categorical variables were compared using the chi-square test or Fisher’s exact test, as appropriate. Univariate analyses were performed to estimate crude odds ratios (ORs) with corresponding 95% confidence intervals (CIs). Variables demonstrating biological plausibility or statistical significance in univariate testing were entered into a multivariable logistic regression model. Model calibration was assessed using the Hosmer-Lemeshow goodness‑of‑fit test, and discrimination was evaluated with the c statistic. Independent predictors of mortality were identified through multivariable logistic regression, with CA-AKI outcome (survivors vs. non‑survivors) specified as the dependent variable. Adjusted ORs with 95% CIs were reported. All tests were two‑sided, and a p-value <0.05 was considered statistically significant.

## Results

Baseline characteristics

Baseline demographic and clinical characteristics of the cohort are presented in Table 1. Among 264 patients with CA‑AKI, nearly half were between 41 and 60 years of age. Fever was the most common presenting symptom, followed by gastrointestinal and respiratory complaints; pedal edema and chest crepitations were the most frequently observed clinical findings. Nearly half of the cohort (126, 47.7%) had a mild comorbidity burden as assessed by the Charlson Comorbidity Index [15].

**Table 1:** Baseline demographic and clinical characteristics of cases of CA-AKI (n=264) CA-AKI: Community-acquired acute kidney infection; COPD: chronic obstructive pulmonary disease

Characteristic	Category	Cases n (%)	95% CI
Gender	Male	172 (65.15)	–
	Female	92 (34.85)	–
Mean age±SD (years)	52.24±15.99		
Age group (years)	18-30	27 (10.23)	–
	31-40	35 (13.26)	–
	41-50	58 (21.97)	–
	51-60	61 (23.11)	–
	61-70	42 (15.91)	–
	71-87	41 (15.53)	–
Clinical presentation of AKI, n (%)	Fever	103 (39.02)	33.1-45.2
	Vomiting	65 (24.62)	19.5-30.3
	Cough	57 (21.59)	16.8-27.0
	Burning micturition	31 (11.74)	8.1-16.3
	Dyspnoea	30 (11.34)	7.8-15.8
	Diarrhoea	22 (8.33)	5.3-12.3
	GI blood loss	18 (6.82)	4.1-10.6
	Loss of Appetite	9 (3.41)	1.6-6.4
	Local pain/swelling (snake bite)	3 (1.14)	0-3.3
	Hematuria	1 (0.38)	0-2.1
Examination findings, n (%)	Edema feet	77 (29.17)	23.8-35.1
	Chest crepitations/rhonchi	76 (28.79)	23.4-34.7
	Signs of dehydration	34 (12.88)	9.1-17.5
	Jaundice	32 (12.12)	8.4-16.7
	Raised JVP	28 (10.61)	7.2-15.0
	Facial puffiness	6 (2.27)	0.1-4.9
	Cyanosis	3 (1.14)	0-3.3
Charlson Comorbidity Index (CCI), n (%)	Mild (1–2)	126 (47.73)	41.6-53.9
	Moderate (3–4)	77 (29.17)	23.8-35.1
	Severe (≥5)	61 (23.11)	18.2-28.7
Comorbidities, n (%)	Hypertension	98 (37.12)	31.3-43.3
	No Comorbidity	78 (29.55)	24.1-35.4
	Diabetes Mellitus	75 (28.41)	23.0-34.3
	Ischemic heart disease	45 (17.05)	12.7-22.1
	Chronic liver disease	40 (15.15)	11.0-20.1
	Stroke	32(12.12)	8.4-16.7
	COPD	13 (4.92)	2.6-8.3
	Connective tissue disease	5 (1.89)	0.1-4.4
	Hepatitis B infection	5 (1.89)	0.1–4.4
	Tuberculosis	4 (1.52)	0-3.8
	HIV	3 (1.14)	0-3.3
	Hemoglobinopathies	3 (1.14)	0-3.3
AKI Stage at presentation, n (%)	Stage 1	157 (59.47)	53.3-65.4
	Stage 2	57 (21.59)	16.8-27.0
	Stage 3	50 (18.94)	14.4-24.2

Etiologies and attributing factors

Infections associated with CA‑AKI are listed in Table 2. Infections were the leading cause, accounting for 142 cases (53.8%). Respiratory infections - including community‑acquired and aspiration pneumonia - were most frequent, followed by urinary tract and gastrointestinal infections. Central nervous system infections and other infectious conditions, such as scrub typhus and cellulitis, were also observed. Non‑infectious causes included heart failure-related AKI (41, 15.2%), nephrotoxic drug exposure (15, 5.7%), and cirrhosis‑related (hepato-renal syndrome) AKI (11, 4.2%).

**Table 2:** Infections associated CA-AKI (n=142) Others included one  case each of neutropenic colitis, abdominal tuberculosis, pyogenic meningitis, infective exacerbation of ILD, lung abscess, pulmonary mucormycosis, tubercular pericardial effusion, infective. COPD: Chronic obstructive pulmonary disease; ILD: interstitial lung disease.

Infections	n (%)
Urinary tract infection	34 (12.9)
Community-acquired pneumonia	22 (8.3)
Aspiration pneumonia	15 (5.7)
Gastroenteritis	11 (4.2)
Pyrexia of unknown origin	8 (3.0)
Infective exacerbation of COPD	7 (2.7)
Tubercular meningitis	5 (1.9)
Pulmonary tuberculosis	5 (1.9)
Viral meningitis	4 (1.5)
Scrub typhus	4 (1.5)
Acute pancreatitis	4 (1.5)
Spontaneous bacterial peritonitis	3 (1.1)
Acute viral hepatitis	3 (1.1)
Lower limb cellulitis	3 (1.1)
Cholecystitis	2 (0.8)
Dengue fever	2 (0.8)
Others	10 (3.8)

The attributing factors for CA-AKI are shown in Table 3. Pre-renal etiologies predominated, accounting for nearly two-thirds of all cases (197, 74.6%). Sepsis was the single most frequent contributor (73, 27.6%), followed by volume depletion (50, 18.9%), cardio-renal syndrome (41, 15.5%), hemorrhage (22, 8.3%), and hepato-renal syndrome (11, 4.2%). Renal causes included infections (18, 6.8%), nephrotoxic drugs (15, 5.7%), and less frequent causes like malignant hypertension, lupus nephritis, endogenous toxins, snakebite, renal artery stenosis, and vasculitis. Post-renal etiologies were relatively uncommon. Overall, pre-renal factors - particularly sepsis - were the dominant contributors, underscoring the importance of systemic and hemodynamic disturbances over obstructive or intrinsic renal pathology in this population.

**Table 3:** Contributing factors to CA-AKI (n=264) *In cases where multiple factors contributed to AKI, the single factor judged by the treating physician to be the most attributable was recorded as the contributing factor. CA-AKI: Community-acquired acute kidney injury.

Category (n, %)	Contributing factor*	Frequency , n (%)
Pre-renal (197, 74.6%)	Sepsis	73 (27.6)
	Volume depletion	50 (18.9)
	Cardiorenal syndrome	41 (15.5)
	Hemorrhage	22 (8.3)
	Hepatorenal syndrome	11 (4.2)
Renal (55, 20.9%)	Infections	18 (6.8)
	Nephrotoxic drugs	15 (5.6)
	Malignant hypertension	9 (3.4)
	Lupus nephritis	5 (1.9)
	Endogenous toxins	4 (1.5)
	Snake bite	2 (0.8)
	Renal artery stenosis	1 (0.4)
	Vasculitis	1 (0.4)
Post-renal (12, 4.5%)	Extrarenal obstruction	11 (4.2)
	Nephrolithiasis	1 (0.4)

Outcomes

Clinical outcomes are shown in Table 4. Of 264 cases, 32 (12.1%) died, while 160 (60.6%) recovered. Early recovery occurred in 101 cases (38.3%; 95% CI, 32.4-44.4), whereas 59 (22.4%; 95% CI, 17.5-27.9) experienced delayed recovery. Progression to AKD was substantial (72, 27.3%), distributed across stages 0 to 3. Although recovery was common, a significant proportion of the patients experienced delayed recovery or progression to AKD. Progression to chronic kidney disease could not be assessed due to the short follow‑up period.

**Table 4:** Outcomes of CA-AKI cases (n=264) CA-AKI: Community-acquired acute kidney infection; AKD: acute kidney disease; CI: confidence interval

Outcome of AKI	Number of subjects	95% CI
Mortality, n (%)	32 (12.12)	8.4-16.7
Early recovery, n (%)	101 (38.26)	32.4-44.4
Delayed recovery, n (%)	59 (22.35)	17.5-27.9
AKD 0, n (%)	14 (5.3)	2.9-8.7
AKD 1, n (%)	19 (7.2)	4.4-11.0
AKD 2, n (%)	15 (5.68)	3.2-9.2
AKD 3, n (%)	24 (9.09)	5.9-13.2

Compared with patients who recovered, those without recovery had significantly higher mean serum urea and creatinine levels (p<0.0001), lower serum sodium concentrations (p=0.008), and higher leukocyte counts (p=0.04). They were more likely to require renal replacement therapy (p<0.0001) and to develop complications, including multi-organ dysfunction syndrome (MODS; p<0.0001), acute respiratory distress syndrome (ARDS; p=0.01), need for ventilatory support (p<0.0001), and encephalopathy (p<0.0001). Similarly, non-survivors differed from survivors by markedly higher rates of MODS (p<0.0001), ventilatory support (p<0.0001), ARDS (p=0.01), and encephalopathy (p<0.0001), whereas baseline comorbidities and most laboratory parameters did not differ significantly between groups (Table 5). Overall, renal replacement therapy was required in 20 patients (7.6%).

**Table 5:** Clinical characteristics, laboratory parameters, complications in recovered vs not recovered and survivors vs non-survivors group of CA-AKI Values are presented as mean±SD for continuous variables and n (%) for categorical variables,  χ²=chi‑square test statistic, t=Student’s t‑test statistic, *Welch’s t-test (unequal variance), Fisher’s=Fisher’s exact test. MODS=Multiple Organ Dysfunction Syndrome, ARDS=Acute Respiratory Distress Syndrome, CCI=Charlson Comorbidity Index.

Category	Recovered (n=159)	Not Recovered (n=105)	Statistical Test, p-value	Non-survivors (n=32)	Survivors (n=232)	Statistical Test, p-value
Comorbidities						
No Comorbidity, n (%)	51 (32.1)	27 (25.7)	χ²=1.22, p=0.27	8 (25.0)	70 (30.2)	χ²=0.35, p=0.55
Hypertension, n (%)	60 (37.7)	38 (36.2)	χ²=0.06, p=0.79	9 (28.1)	89 (38.4)	χ²=1.21, p=0.27
Diabetes Mellitus, n (%)	39 (24.5)	36 (34.3)	χ²=2.95, p=0.08	12 (37.5)	63 (27.2)	χ²=1.45, p=0.23
Ischemic Heart Disease, n (%)	20 (12.6)	25 (23.8)	χ²=5.65, p=0.01	9 (28.1)	36 (15.5)	χ²=2.90, p=0.09
Stroke, n (%)	22 (13.8)	10 (9.5)	χ²=1.11, p=0.29	4 (12.5)	28 (12.1)	χ²=0.00, p=0.96
Chronic Liver Disease, n (%)	23 (14.5)	17 (16.2)	χ²=0.15, p=0.70	8 (25.0)	32 (13.8)	χ²=2.28, p=0.13
Laboratory Parameters (mean±SD)						
Urea (mg/dL)	66.9±28.0	88.1±54.3	t=3.90, p<0.0001	85.1± 50.6	74± 40.3	t=1.27, p=0.21
Creatinine (mg/dL)	1.9±0.7	3.3±3.2	t=4.25, p<0.0001	2.9± 3.3	2.4± 2.0	t=0.97, p=0.33
Serum Sodium (mEq/L)	137.9±7.1	135.5±7.7	t=2.64, p=0.008	135.4± 8.7	137.2± 8.3	t=-1.09, p=0.28
Serum Potassium (mEq/L)	4.2±0.8	4.3±0.8	t=1.06, p=0.29	4.2± 0.8	4.3± 0.8	t=-0.63, p=0.53
Total Leukocyte Count (×10³/mm³)	10788.4±6072.8	13906.4±18255.3	t=2.00, p=0.04	12597.2± 4853.1	11950± 13217.8	*t=0.27, p=0.79
Hemoglobin (g/dL)	10.4±2.9	10.1±2.4	t=0.88, p=0.38	9.6± 2.6	10.4± 2.8	t=-1.55, p=0.12
Platelets (×10³/µL)	184459.1±106886.9	181742.9±92703.5	t=0.21, p=0.83	166750± 87685.2	185672.4± 103004.3	t=-1.01, p=0.31
Complications During Hospital Stay						
Renal Replacement Therapy, n (%)	0 (0.0)	20 (19.1)	Fisher’s, p<0.0001	5 (15.6)	15 (6.5)	χ²=2.85, p=0.09
MODS, n (%)	8 (5.0)	37 (35.2)	χ²=38.2, p<0.0001	21 (65.6)	24 (10.3)	χ²=52.6, p<0.0001
ARDS, n (%)	2 (1.3)	7 (6.7)	Fisher’s, p=0.01	4 (12.5)	5 (2.2)	Fisher’s, p=0.01
Anemia, n (%)	40 (25.2)	40 (38.1)	χ²=5.00, p=0.02	8 (25.0)	72 (31.0)	χ²=0.47, p=0.49
Need for Ventilator Support, n (%)	18 (11.3)	32 (30.5)	χ²=17.9, p<0.0001	26 (81.2%	24 (10.3)	χ²=84.6, p<0.0001
Encephalopathy, n (%)	18 (11.3)	31 (29.5)	χ²=15.1, p<0.0001	19 (59.4)	30 (12.9)	χ²=38.7, p<0.0001
Charlson Comorbidity Index (CCI)						
Mild (1-2), n (%)	79 (49.7)	47 (44.8)	χ²=0.41, p=0.52	13 (40.6)	113 (48.7)	χ²=0.77, p=0.38
Moderate (3-4), n (%)	47 (29.6)	30 (28.6)	χ²=0.41, p=0.52	8 (25.0)	69 (29.7)	χ²=0.26, p=0.61
Severe (≥ 5), n (%)	33 (20.7)	28 (26.6)	χ²=0.41, p=0.52	11 (34.4)	50 (21.6)	χ²=2.28, p=0.13

Predictors of mortality

Table 6 summarizes the findings of the multivariate logistic regression analysis evaluating predictors of mortality in patients with community‑acquired AKI. Among the variables assessed, MODS, need for ventilatory support, and encephalopathy were identified as independent predictors of death, with adjusted odds ratios of 4.56 (95% CI, 1.33-15.66; p=0.016), 17.3 (95% CI, 5.22-57.19; p=0.001), and 6.32 (95% CI, 1.68-23.83; p=0.006), respectively. Other factors - including age, Glasgow Coma Scale category, Charlson Comorbidity Index, dyspnea, volume depletion, cirrhosis, and ARDS - did not reach statistical significance.

**Table 6:** Predictors of mortality in CA-AKI cases (Multivariate logistic regression analysis) *Wald chi‑square. CA-AKI: Community-acquired acute kidney disease; ARDS: acute respiratory distress syndrome; OR: odds ratio.

Predictor	Adjusted OR	95% CI for OR	Test (statistic)	p‑value
Age (per year increase)	0.96	0.92-1.01	Wald χ²=2.60	0.11
Charlson Comorbidity Index (CCI) category	1.55	0.71-3.41	Wald χ²=1.20	0.27
Dyspnea	3.71	0.86-16.01	Wald χ²=3.12	0.07
Volume depletion	0.53	0.06-4.51	Wald χ²=0.34	0.56
Cirrhosis	1.73	0.24-12.25	Wald χ²=0.30	0.58
MODS (multi‑organ dysfunction syndrome)	4.56	1.33-15.66	Wald χ²=5.80	0.01
ARDS	0.3	0.04-2.21	Wald χ²=1.40	0.23
Need for ventilator	17.3	5.22-57.19	Wald χ²=10.90	0.001
Encephalopathy	6.32	1.68-23.83	Wald χ²=7.60	0.006

## Discussion

This prospective study provides a comprehensive evaluation of the demographic, clinical, and etiological characteristics of patients with CA-AKI in a tertiary care center in Central India.

Etiology and clinical characteristics

Infections emerged as the predominant etiology, accounting for more than half of all cases (142, 53.8%), consistent with prior Indian studies [16,17]. This contrasts sharply with data from developed countries, where nephrotoxic drugs and chronic comorbidities such as diabetes and hypertension are more frequently implicated [18]. Respiratory, urinary tract, and gastrointestinal infections were the most frequent contributors, underscoring the persistent burden of infectious diseases in this region.

Our findings align with reports from Eastern and Southern India that highlight regional variation in AKI etiology - snakebite and toxic exposures in some areas, infections in others [9-11,16,17]. The predominance of infections in Central India underscores the need for region-specific preventive strategies, including early recognition and treatment of sepsis, aggressive fluid resuscitation, and timely referral for renal replacement therapy. The infection-driven burden of AKI reflects broader public health challenges, including inadequate sanitation, high population density, and limited access to timely healthcare. Addressing these systemic issues is critical to reducing the incidence of infection-related AKI.

The clinical profile of our cohort - predominantly middle-aged men with comorbid hypertension, diabetes, and chronic liver disease - illustrates the dual burden of communicable and non-communicable diseases in India. Male predominance (172, 65.2%) is consistent with earlier Indian reports [9,16,17]. Sociocultural factors, including differential healthcare-seeking behavior and occupational exposures, may contribute to this disparity. Men engaged in agriculture and industry may face greater exposure to nephrotoxic substances, increasing their risk of AKI.

Pre-renal AKI was the most common subtype, highlighting the role of preventable factors such as dehydration and hemorrhage. In contrast, hospital-acquired AKI in other settings is more often associated with drug toxicity and complex interventions [8,19], underscoring the distinct epidemiology of CA-AKI. Volume depletion was the most frequent cause and was strongly associated with recovery, reflecting its reversible nature when promptly recognized and corrected. By contrast, hepatorenal syndrome, though less common, was significantly associated with non-recovery, underscoring its poor prognosis and limited reversibility in advanced liver disease [20]. Other etiologies - including congestive cardiac failure, hemorrhage, infections, nephrotoxic drugs, and renal ischemia - were observed across both recovery and non-recovery groups without statistically significant differences, suggesting that outcomes depend more on severity and timeliness of intervention than on the underlying cause. These findings emphasize the importance of early identification of volume depletion as a modifiable risk factor, while recognizing hepatorenal syndrome as a marker of poor prognosis.

Impact of AKI stage and type

Severity at presentation was striking, with 40.5% (n=107) of patients presenting with Stage 2 or Stage 3 AKI. This finding parallels the International Society of Nephrology-Acute Kidney Injury (ISN-AKI) registry study in India, which reported 54.8% of 3,711 CA-AKI patients presenting with Stage 3 disease [9]. Such patterns suggest delayed diagnosis and treatment, likely driven by limited awareness, socioeconomic barriers, and restricted access to healthcare. Patients with Stage 3 AKI demonstrated significantly lower recovery rates compared with those at earlier stages (p<0.0001), reinforcing the importance of early detection and intervention. Although mortality trended higher in advanced stages, statistical significance was not reached, indicating that outcomes are influenced by multifactorial determinants beyond AKI stage alone.

Pre-renal AKI, caused by renal hypoperfusion rather than tubular injury, typically demonstrates rapid recovery once perfusion is restored [21]. This underscores the prognostic importance of etiology and timely intervention, particularly in pre-renal AKI.

Predictors of recovery and mortality

The overall recovery in our cohort was 60.6% (160/264), lower than the 80.8% recovery rate reported by Peerapornratana et al. in their long-term study [22]. Differences in demographic factors, comorbidities, and stage/type of AKI at presentation likely contributed to this variation. Mortality in our study was 12.1% (32/264), comparable to the multi-center prospective cohort study by Prasad et al. (n=3711), which reported an index admission mortality of 10.8% [9].

Multivariate analysis identified MODS, ventilator requirement, and encephalopathy as independent predictors of mortality. The strong association between ventilator support and death (adjusted OR, 17.3) underscores the prognostic weight of respiratory failure and systemic illness severity. Encephalopathy (adjusted OR, 6.32) reflects advanced metabolic derangements and central nervous system involvement, both of which portend poor outcomes. MODS (adjusted OR, 4.56) highlights the cumulative impact of multi-system involvement, consistent with prior studies linking organ failure burden to mortality in critical illness. By contrast, demographic factors such as age and comorbidity burden did not independently predict outcome, suggesting that acute physiological deterioration outweighed baseline risk in determining survival.

While uremic toxin accumulation, metabolic acidosis, electrolyte derangements, and fluid overload remain well-recognized contributors to mortality in AKI, accumulating evidence highlights that severe AKI is best understood as a systemic disease [23]. Increasingly, AKI is implicated in dysfunction of distant organs, including the lung, heart, brain, liver, and intestine, mediated through maladaptive organ-organ communication [24]. The proposed mechanisms for this “AKI-induced distant organ crosstalk” include immune dysregulation, heightened systemic inflammation, endothelial injury, cellular apoptosis, and oxidative stress [25]. These pathways extend the impact of AKI beyond the kidney itself, amplifying multi-organ failure and thereby contributing substantially to excess mortality in critically ill patients.

Clinical and public health implications

Taken together, our results demonstrate that infection-related CA-AKI, delayed presentation, and advanced disease stages remain major challenges in India. Mortality is driven less by AKI stage itself than by systemic complications such as MODS, sepsis, and encephalopathy. These findings call for a dual strategy: strengthening public health measures to reduce infection-related AKI and enhancing clinical systems to enable earlier recognition and intervention. Improved access to primary care, patient education, and aggressive management of complications are essential to improving outcomes in resource-limited settings.

Clinically, this study demonstrates that CA-AKI in Central India is largely infection-related and potentially preventable. Early intervention in reversible causes such as volume depletion and sepsis is critical, while patients with systemic complications such as MODS or hepatorenal syndrome represent high-risk groups requiring intensive management. These findings provide evidence to guide clinical practice and inform public health strategies aimed at reducing the burden of CA-AKI in resource-limited settings.

Limitations

This study has several limitations. First, it was conducted at a single tertiary care center in Central India, which may restrict the generalizability of the findings to other settings with different epidemiologic patterns of acute kidney injury. Second, follow‑up was limited to the duration of hospitalization, precluding evaluation of long‑term outcomes such as progression to CKD. Third, although standardized KDIGO criteria were applied, the observational design may have introduced residual confounding, particularly in the attribution of etiologies. Fourth, surgical causes of AKI were not included, narrowing the scope of etiological representation. In addition, potential selection bias inherent to a tertiary care population, information bias from retrospective data collection, and the risk of overfitting in the multivariable model must be acknowledged. External validation was not performed. Taken together, these limitations underscore the need for multicenter studies with extended follow‑up and independent validation to confirm and extend our findings.

Strengths

The prospective design, reliable follow-up during hospitalization, and use of uniform KDIGO criteria for diagnosis and staging of AKI are major strengths of this study. These features enhance the validity of the findings and provide robust evidence on the epidemiology and outcomes of CA-AKI in Central India.

## Conclusions

Infections - most notably respiratory and urinary tract infections - emerged as the predominant causes of CA‑AKI in our cohort, highlighting the persistent burden of infection‑related AKI in Central India. Patients with pre‑renal AKI and those presenting at stage 1 AKI were more often associated with better renal recovery, underscoring the potential reversibility of early disease when promptly recognized. By contrast, the occurrence of complications such as MODS, ARDS, and encephalopathy was associated with poorer outcomes and heightened mortality. These findings, derived from a prospective observational study, emphasize associations rather than direct causal linkages and underscore the importance of early recognition and prevention strategies in mitigating the burden of CA‑AKI.

Sepsis and dehydration remain dominant etiologies in this region, and their interaction with comorbid conditions amplifies risk. Addressing these challenges will require strengthening public health measures to curb infection‑related AKI, expanding access to primary care, and ensuring aggressive management of complications.

Finally, given the regional variability in etiologies and outcomes, multicenter studies across diverse settings are essential for validating these observations and guiding preventive and therapeutic strategies tailored to resource‑limited environments.
